# Hyperglycemia potentiates increased *Staphylococcus aureus* virulence and resistance to growth inhibition by *Pseudomonas aeruginosa*

**DOI:** 10.1128/spectrum.02299-23

**Published:** 2023-11-07

**Authors:** Christopher J. Genito, Benjamin P. Darwitz, Matthew A. Greenwald, Matthew C. Wolfgang, Lance R. Thurlow

**Affiliations:** 1 Division of Oral and Craniofacial Health Sciences, University of North Carolina at Chapel Hill Adams School of Dentistry, Chapel Hill, North Carolina, USA; 2 Department of Microbiology and Immunology, University of North Carolina at Chapel Hill School of Medicine, Chapel Hill, North Carolina, USA; 3 Marsico Lung Institute, University of North Carolina at Chapel Hill School of Medicine, Chapel Hill, North Carolina, USA; Griffith University - Gold Coast Campus, Gold Coast, Queensland, Australia

**Keywords:** diabetes, indwelling medical devices, glycolysis, USA300, dissemination

## Abstract

Diabetes is associated with several health consequences, including increased susceptibility to more frequent and severe infections. Bacterial infections associated with diabetes are typically polymicrobial, with *Staphylococcus aureus* and *Pseudomonas aeruginosa* frequently isolated from the same infection site. *S. aureus* and *P. aeruginosa* are frequently found in diabetic skin and soft tissue infections, in the lungs of people with cystic fibrosis, and in indwelling device infections. Numerous studies have investigated interactions between these two pathogens primarily using *in vitro* systems. These models have several limitations as they do not accurately reflect the complexities of an immune response nor the nutrient dynamics in a diabetic infection microenvironment. Here, we describe a novel murine indwelling device co-infection model that allows us to study the interactions between *S. aureus* and *P. aeruginosa* within the context of an immune response during both normal and diabetic infections. Our data shows that *P. aeruginosa* significantly inhibits *S. aureus* growth during co-infection in a normal mouse and that inhibition is not dependent on the *P. aeruginosa* PQS quorum sensing system. Conversely, in a diabetic co-infection, *S. aureus* overcomes inhibition by *P. aeruginosa* and this phenotype is reliant on *S. aureus* glycolysis. We also demonstrate that both organisms display increased virulence potential in a diabetic co-infection as we observe increased dissemination to peripheral tissues. This study revealed novel *in vivo* interactions between *S. aureus* and *P. aeruginosa* and advances our understanding of the complex interactions between microorganisms in polymicrobial infections in clinically relevant infection microenvironments.

**IMPORTANCE:**

Individuals with diabetes are prone to more frequent and severe infections, with many of these infections being polymicrobial. Polymicrobial infections are frequently observed in skin infections and in individuals with cystic fibrosis, as well as in indwelling device infections. Two bacteria frequently co-isolated from infections are *Staphylococcus aureus* and *Pseudomonas aeruginosa*. Several studies have examined the interactions between these microorganisms. The majority of these studies use *in vitro* model systems that cannot accurately replicate the microenvironment of diabetic infections. We employed a novel murine indwelling device model to examine interactions between *S. aureus* and *P. aeruginosa*. Our data show that competition between these bacteria results in reduced growth in a normal infection. In a diabetic infection, we observe increased growth of both microbes and more severe infection as both bacteria invade surrounding tissues. Our results demonstrate that diabetes changes the interaction between bacteria resulting in poor infection outcomes.

## INTRODUCTION

Nearly 30 million adults in the United States have clinically diagnosed diabetes, while an additional 130 million individuals are estimated to have undiagnosed diabetes or prediabetes (1). Although diabetes is associated with numerous health complications, individuals with diabetes have increased susceptibility to deep tissue infections, and infections resulting from indwelling medical devices (2 – 4). Diabetic infections are typically recurrent, especially in cases of poorly managed hyperglycemia, and often necessitate limb amputation resulting in significant disability (5 – 9). In healthy individuals, functional innate and adaptive immune responses are critical for repelling pathogens that cause infection. Diabetes confers a state of immunosuppression that contributes to developing ulcerated tissue, typically on the lower extremities (5, 6, 9, 10). Many bacteria are associated with diabetic infections; however, *Staphylococcus aureus* and *Pseudomonas aeruginosa* are frequently isolated from the same infection site (5, 7 – 12) and are among the most common species associated with indwelling medical device infections (13). Once established, these pathogens can penetrate into underlying tissues and subsequently enter the circulatory system, where they disseminate to other areas of the body and cause secondary infections, including infective endocarditis and osteomyelitis (5 – 10).

*S. aureus* secretes multiple virulence factors that cause disease by breaking down host tissues and allow *S. aureus* to evade the host immune response, including multiple proteases, phenol-soluble modulins, pore-forming toxins, and leukocidins (14). Multiple studies demonstrate that *S. aureus* exhibits increased bacterial burden and virulence in diabetic infections compared to infection in individuals without diabetes (5 – 10). *S. aureus* fuels virulence factor production by readily metabolizing glucose obtained via its four dedicated glucose transporters, GlcA, GlcB, GlcC, and GlcU (10, 15), to generate adenosine 5′-triphosphate (ATP) through aerobic respiration or fermentative pathways, depending on environmental nutrient availability (16). During growth in hyperglycemic conditions, *S. aureus* simultaneously fluxes glucose through aerobic respiration and fermentative pathways to maximize ATP production and balance redox stress, known as overflow metabolism (10, 15, 16). This expanded glucose import potential compared to other *Staphylococcus* species, and a glycolytic-dependent mechanism by which *S. aureus* can resist host immune factors, both support the idea that *S. aureus* has evolutionarily adapted to have increased pathogenicity in humans (15, 17).

In contrast, the pathogenicity of *P. aeruginosa* is often attributed to its high level of adaptability. In its native environment of water and soil, *P. aeruginosa* is in constant competition with other microbial species for nutrients and resources (18 – 20). As a result, *P. aeruginosa* has evolved various mechanisms to suppress the growth or otherwise kill bacterial competitors by secreting factors that inhibit bacterial aerobic respiration such as pyocyanin, 2-heptyl-4-hydroxyquinoline-*N*-oxide (HQNO), and hydrogen cyanide (HCN) (20, 21). *P. aeruginosa* encodes three interconnected quorum sensing (QS) systems, PQS, Rhl, and Las, that control the production of several factors that are known to inhibit *S. aureus* growth (22, 23). The main transcriptional regulator of the PQS system, PqsR (MvfR), directly contributes to the control of the transcriptional regulators of the Rhl and Las QS systems, RhlR and LasR, respectively (24). Previous studies demonstrate that deletion of *pqsR* results in abolished production of HQNO and pyocyanin (24 – 26). Additionally, PqsR is known to indirectly contribute to the production of hydrogen cyanide, elastase, rhamnolipids, and LasA protease, which are additional factors that allow *P. aeruginosa* to compete with other bacterial species, as well as contribute directly to disease in humans (26).

In this study, we examine growth and tissue dissemination of *S. aureus* and *P. aeruginosa* during mono- and co-infection of an indwelling catheter in healthy and diabetic mice. Our lab has developed a novel murine co-infection model that utilizes a previously described subcutaneous catheter insertion model (27), followed by sequential inoculation with *S. aureus* and later with *P. aeruginosa*. This sequential inoculation allows *S. aureus* to first establish infection in order to examine how *P. aeruginosa* influences *S. aureus* behavior.

Many labs use *in vitro* models to study the interactions between *S. aureus* and *P*. *aeruginosa*. However, a significant drawback to these studies is that they are in a closed system in artificial, nutrient-limiting conditions and outside of the context of the complex host immune response. Currently, few models exist that replicate host conditions during multispecies infections. Therefore, it is imperative to develop a model that accurately reflects the dynamics of *S. aureus* and *P. aeruginosa* in the context of diabetic infections that are not available in traditional *in vitro* systems. Unlike traditional *in vitro* culture conditions, our murine co-infection catheter model sustains chronic bacterial infections where nutrients are continually replenished by the host circulatory system. In addition to recapitulating infection of indwelling medical devices, this model allows us to observe these bacteria more generally within the complex infection microenvironment of diabetic tissues and in the context of a host immune response.

In addition to indwelling catheters, the interaction of the dominant pathogens, *P. aeruginosa* and *S. aureus*, is relevant to lung disease in patients with cystic fibrosis (CF) (21, 28 – 30). Typically, *S. aureus* first colonizes the lungs of children with CF and is later replaced by *P. aeruginosa* as the dominant pathogen during adolescence and adulthood (21, 30, 31). Another significant advantage to the catheter model is that we can inoculate the catheters sequentially with *S. aureus* and *P. aeruginosa* to mimic the dynamics of temporal infection typical of diabetic wounds and CFRD lung infections. A common co-morbidity of CF is the development of CF-related diabetes (CFRD). Interestingly, *S. aureus* and *P. aeruginosa* display an altered dynamic in the lungs of patients with CFRD, wherein *S. aureus* re-emerges as the dominant pathogen in the airways of CF patients after initially being replaced by *P. aeruginosa* (28, 32). Furthermore, skin and soft tissue infections (SSTIs) with *P. aeruginosa* and *S. aureus* are a significant source of morbidity in individuals with diabetes (5 – 9, 11, 12, 33). These observations raise several important questions about the dynamics between *S. aureus* and *P. aeruginosa* and the metabolic interplay that results in altered interactions in diabetic infections. Although the pathogenicity and virulence mechanisms of *S. aureus* and *P. aeruginosa* are the subject of numerous studies, there remains a significant knowledge gap defining how *S. aureus* and *P. aeruginosa* interact *in vivo* and how infection dynamics change during diabetic co-infections.

In this study, we show that *S. aureus* growth is inhibited during co-infection with *P. aeruginosa* but significantly less inhibited during a diabetic infection. Additionally, we observe that both *S. aureus* and *P. aeruginosa* virulence is more severe in a diabetic host via increased dissemination into the tissue. We observed that *P. aeruginosa* growth is moderately inhibited during co-infection with *S. aureus*, which is mediated by both glucose-dependent and -independent mechanisms depending on the metabolic state of the host environment. We provide evidence that *P. aeruginosa* PqsR-regulated secreted factors are responsible for growth inhibition of *S. aureus in vitro,* and glucose availability and glycolytic function are required for *S. aureus* resistance to *P. aeruginosa*-mediated inhibition *in vitro* and *in vivo*. Interestingly, we demonstrate that *P. aeruginosa* inhibition of *S. aureus* growth during co-infection *in vivo* does not require PqsR-regulated factors and is instead mediated by unknown factors. Taken together, we demonstrate that our *in vivo* catheter model can reveal novel interactions between *S. aureus* and *P. aeruginosa*, as well as elucidate the virulence potential of these bacterial pathogens that are masked in traditional *in vitro* systems.

## RESULTS

### *S. aureus* proliferation during catheter infection is inhibited by *P. aeruginosa*

**Fig 1:**
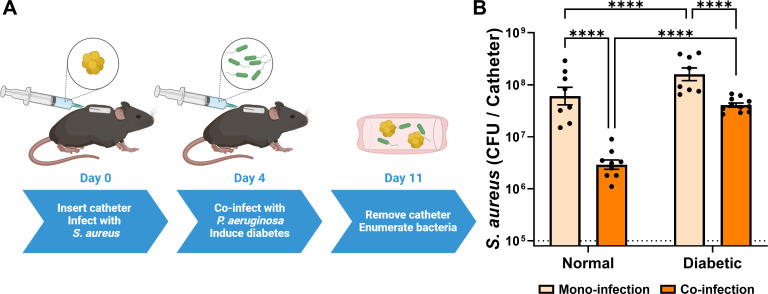
Inhibitive effects of *P. aeruginosa* on *S. aureus* during co-infection are lessened in a diabetic host. (**A**) A schematic of the diabetic catheter infection model used throughout this study. (**B**) *S. aureus* colony-forming units (CFU) recovered from the catheter 11 days after initial infection, either alone or in co-infection with *P. aeruginosa*. Infections were carried out in mice treated with streptozotocin (diabetic) or untreated mice (normal). Bars represent geometric mean and standard error. Dotted line represents 10^5^ CFU catheter inoculum. ANOVA with Tukey’s test for multiple comparisons: ****P < 0.0001.

To determine the proliferative potential of *S. aureus* in the presence of *P. aeruginosa* during catheter co-infection, subcutaneous catheters were infected with *S. aureus* (1 × 10^5^ CFU) alone or sequentially co-infected with *S. aureus* (1 × 10^5^ CFU) and 4 days later with *P. aeruginosa* (1 × 10^5^ CFU) (Fig. 1). During mono-infection, *S. aureus* burden increased 2–3.5 logs by day 11 post-infection. However, *S. aureus* burden within the catheter was significantly reduced (~20 fold on average, *P* < 0.0001) by day 11 when catheters were subsequently co-infected with *P. aeruginosa*.

### The inhibitory effect of *P. aeruginosa* on *S. aureus* is lessened in a diabetic host

Co-infection of *S. aureus* and *P. aeruginosa* using a subcutaneous catheter was also performed in diabetic mice where diabetes was induced with streptozotocin at day four (Fig. 1). Diabetes was induced at day four to ensure that there was a similar amount of *S. aureus* in all catheters when *P. aeruginosa* was introduced. In mono-infection, *S. aureus* grew to a statistically higher burden in an infected catheter within diabetic mice than within normal mice (~3 fold, *P* < 0.0001). More importantly, *S. aureus* was able to grow to a significantly higher burden in the presence of *P. aeruginosa* in a diabetic host than in a normal host, >10 fold (*P* < 0.0001) the average observed in the catheter of normal mice during co-infection. These experiments establish that *S. aureus* is able to overcome growth inhibition by *P. aeruginosa* during co-infection in a diabetic host environment.

### Inhibition of *S. aureus* growth by *P. aeruginosa* is ameliorated by glucose availability *in vitro*

**Fig 2:**
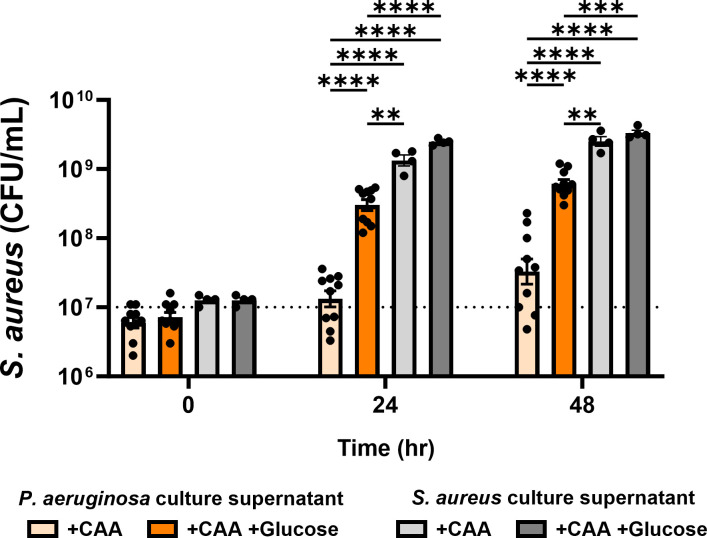
The *S. aureus* growth inhibition by *P. aeruginosa* secreted factors is ameliorated by glucose availability. *S. aureus* was cultured *in vitro* in the presence of *P. aeruginosa* or *S. aureus* culture supernatant supplemented with or without 25 mM glucose. All supernatants were fortified with 1% casamino acids before inoculation with *S. aureus*. Supernatants without glucose were supplemented with an additional carbon-equivalent of casamino acids. Bars represent geometric mean and standard error. Dotted line represents 10^7^ CFU/ml culture inoculum. ANOVA with Tukey’s test for multiple comparisons: **P < 0.01, ***P < 0.001, ****P < 0.0001.

Given the reduction of *S. aureus* growth observed *in vivo* when co-infecting with *P. aeruginosa*, we suspected that *P. aeruginosa* secreted factors may be responsible for *S. aureus* growth inhibition as indicated by previous studies (28, 30, 34, 35). To model *S. aureus* inhibition by *P. aeruginosa* secreted factors *in vitro*, *S. aureus* was grown in purified spent *P. aeruginosa* culture supernatants, where the supernatant from an overnight culture of *P. aeruginosa* was filter sterilized and fortified with casamino acids (CAA) before the addition of *S. aureus. S. aureus* grown in *P. aeruginosa* culture supernatant showed minimal growth over 24–48 h incubation, significantly less than when grown in purified spent *S. aureus* culture supernatant (Fig. 2). We established in our *in vivo* model that more glucose was available in the catheter environment of diabetic mice than in the catheter environment of normal mice (Fig. S1). We, therefore, hypothesized that the hyperglycemic environment of the diabetic host was responsible for *S. aureus* resistance to *P. aeruginosa* growth inhibition. We used purified spent *P. aeruginosa* supernatants further fortified with glucose and an equimolar amount of CAA to grow *S. aureus in vitro*. In our *in vitro* model, we observed significantly higher *S. aureus* growth in *P. aeruginosa* culture supernatant when the supernatant was supplemented with glucose and CAA compared to supernatant supplemented with only CAA. These experiments provided evidence that the increased glucose availability in a diabetic environment could confer resistance to *S. aureus* growth inhibition by *P. aeruginosa* secreted factors.

### Glycolysis is required for resistance of *S. aureus* to *P. aeruginosa* soluble factors *in vitro*

**Fig 3:**
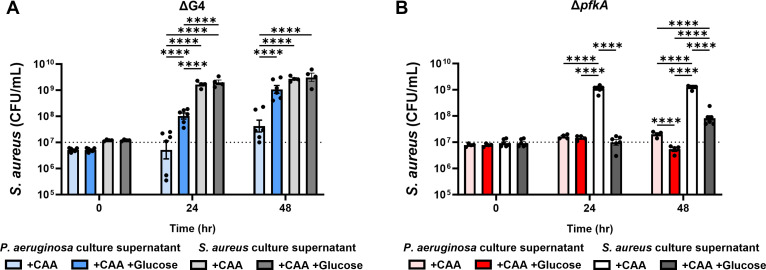
Glycolysis is required for resistance of *S. aureus* to *P. aeruginosa* soluble factors. Mutant *S. aureus* lacking gene(s) for (**A**) four glucose transporters (ΔG4) or (**B**) 6-phosphofructokinase (Δ*pfkA*) was cultured *in vitro* in the presence of *P. aeruginosa* or wild-type *S. aureus* culture supernatant supplemented with or without 25 mM glucose. All supernatants were fortified with 1% casamino acids (CAA) before inoculation with *S. aureus*. Supernatants without glucose were supplemented with an additional carbon-equivalent of casamino acids. Bars represent geometric mean and standard error. Dotted line represents 10^7^ CFU/mL culture inoculum. ANOVA with Tukey’s test for multiple comparisons: ****P < 0.0001.

Further mechanistic analysis of how *S. aureus* glucose metabolism confers resistance to growth inhibition by *P. aeruginosa* secreted factors was performed using two isogenic *S. aureus* mutant strains: (a) an *S. aureus* strain lacking four glucose transporters (ΔG4), which decreases, but does not inhibit glucose uptake altogether (15), and (b) an *S. aureus* strain lacking the gene for 6-phosphofructokinase (Δ*pfkA*) and, therefore, unable to run glycolysis after conversion of glucose 6-phosphate to fructose 6-phosphate (17). As observed with WT *S. aureus*, the ΔG4 mutant showed <1 log growth in *P. aeruginosa* culture supernatant over a 24–48 h period, and supplementation of the supernatant with glucose allowed significantly higher growth (Fig. 3A). The growth of the Δ*pfkA S. aureus* mutant, however, was significantly inhibited by *P. aeruginosa* culture supernatant, both with and without the supplementation of glucose (Fig. 3B). Interestingly, Δ*pfkA* growth was significantly inhibited by the presence of glucose, even when grown in spent *S. aureus* culture supernatant. Given that the Δ*pfkA* mutant has the machinery to import glucose but not fully metabolize it, we suspect that an accumulation of intracellular fructose-6-phospate induces cell death (36). From these observations, we concluded that slower glucose uptake by *S. aureus* did not impact resistance to *P. aeruginosa* soluble factors over a 24–48 h period, but that functional glycolysis was required for resistance.

### Glycolysis supports *S. aureus* growth in catheter infection and resistance to *P. aeruginosa*-mediated inhibition

**Fig 4:**
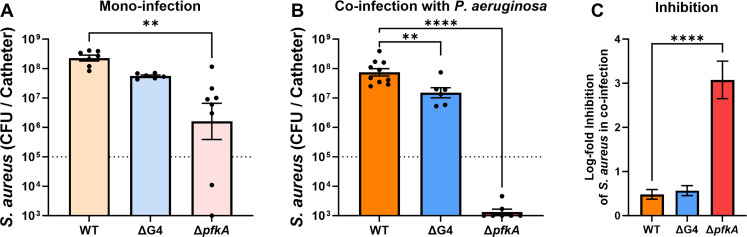
Glycolysis is required for *S. aureus* resistance to *P. aeruginosa* growth inhibition during catheter co-infection in diabetic mice. Wild-type, ΔG4, or Δ*pfkA S. aureus* colonies recovered from catheters inserted into diabetic mice 11 days after infection, either (**A**) alone or (**B**) co-infected with *P. aeruginosa*. (**C**) Log-transformed difference in recovered *S. aureus* CFU from the catheter between mono-infection and co-infection with *P. aeruginosa*, representing inhibited growth by *P. aeruginosa.* Bars represent geometric mean and standard error. Dotted line represents 10^5^ CFU catheter inoculum. ANOVA with Dunnett’s test for multiple comparisons (to WT): **P < 0.01, ****P < 0.0001.

To further test our hypothesis that the hyperglycemic environment of the diabetic host confers resistance of *S. aureus* to *P. aeruginosa*-mediated growth inhibition, analysis of glucose uptake and glycolytic *S. aureus* mutants was performed in co-infection with *P. aeruginosa* in the catheter infection model in diabetic mice (Fig. 4). During mono-infection, the burden of the ΔG4 mutant within the catheter was not significantly different than WT *S. aureus* (Fig. 4A). In contrast, the burden of the Δ*pfkA* mutant was significantly less than WT *S. aureus* (*P* < 0.0001), ~2 log-fold on average. The magnitude of this difference was greatly increased in co-infection with *P. aeruginosa*, with the Δ*pfkA S. aureus* mutant displaying a significant ~5 log-fold reduction in burden compared to WT *S. aureus* (Fig. 4B). The ΔG4 *S. aureus* mutant showed significantly less burden compared to WT *S. aureus* during co-infection with *P. aeruginosa* (~5 fold). This difference was minimal compared to the difference in burden during mono-infection (~4 fold). The magnitude by which *P. aeruginosa* inhibited ΔG4 *S. aureus* mutant growth during co-infection was not significantly different from WT SA growth inhibition by *P. aeruginosa* (Fig. 4C). In contrast, the Δ*pfkA S. aureus* mutant was >1,000 fold more sensitive than WT *S. aureus* to growth inhibition by *P. aeruginosa* during co-infection. Importantly, the Δ*pfkA S. aureus* mutant burden during co-infection with *P. aeruginosa* was significantly below inoculum (~2 log-fold, *P <* 0.0001) and significantly less than the burden in mono-infection (~3 log-fold, *P =* 0.0005), suggesting substantial growth inhibition of the Δ*pfkA S. aureus* mutant by *P. aeruginosa* during co-infection. In normal mice, we again observed that the Δ*pfkA S. aureus* mutant had significantly less growth than WT *S. aureus*, both in mono-infection and during co-infection with *P. aeruginosa* (Fig. S2AB). However, we did not observe a significant sensitivity of the Δ*pfkA S. aureus* mutant to *P. aeruginosa*-mediated growth inhibition during co-infection in normal mice (Fig. S2C). From these *in vivo* experiments, glycolysis was determined to be a factor in both the success of *S. aureus* catheter infection and the resistance of *S. aureus* to growth inhibition by *P. aeruginosa* under diabetic conditions.

### Dissemination of both *S. aureus* and *P. aeruginosa* from catheter infection into the tissue is heightened in a diabetic host

**Fig 5:**
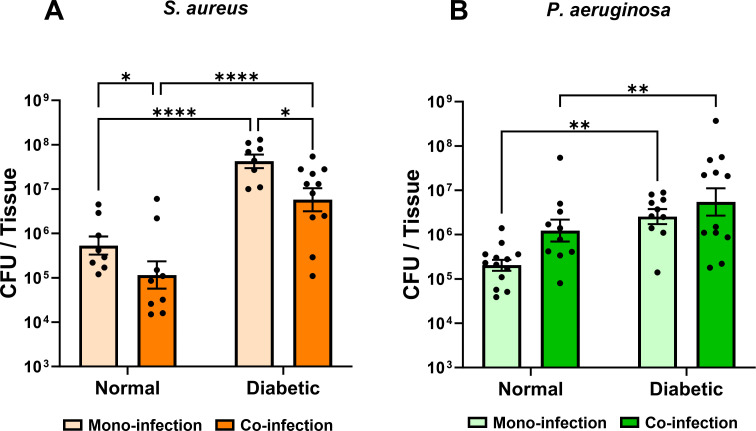
Diabetes supports both *S. aureus* and *P. aeruginosa* dissemination into tissues. (**A**) *S. aureus* or (**B**) *P. aeruginosa* colonies recovered from the surrounding tissue 11 days after catheter insertion and infection, either alone or in co-infection. Infections were carried out in normal or diabetic mice. Bars represent geometric mean and standard error. ANOVA with Tukey’s test for multiple comparisons: *P < 0.05, **P < 0.001, ****P < 0.0001.

We observed that *S. aureus* mono-infection of the catheter in normal mice led to substantial dissemination of *S. aureus* into the tissue surrounding the catheter (Fig. 5A). *S. aureus* dissemination into the tissue was increased ~2 log-fold in diabetic mice. Co-infection in normal mice with *P. aeruginosa* significantly inhibited *S. aureus* dissemination into the tissues, though only moderately in either normal or diabetic mice. We concluded that, in addition to an effect on growth within the catheter, *P. aeruginosa* had an inhibitive effect on *S. aureus* dissemination into the tissue during catheter co-infection. However, while the diabetic environment enhanced *S. aureus* dissemination into the tissue, it did not affect the inhibitive ability of *P. aeruginosa* on *S. aureus* dissemination.

We also observed that the diabetic host environment supported modest, but significant dissemination of *P. aeruginosa* into the tissue during catheter infection (Fig. 5B). There also was a trend that co-infection with *S. aureus* moderately increased *P. aeruginosa* dissemination into the tissue during infection in normal mice, including in co-infections with ΔG4 and Δ*pfkA* mutant *S. aureus* (Fig. S3). Ultimately, it was concluded that the diabetic host environment increased both *S. aureus* and *P. aeruginosa* dissemination into the tissue during catheter infection in both mono- and co-infection.

### *S. aureus* glycolysis is required for increased dissemination into tissues during diabetic infection

**Fig 6:**
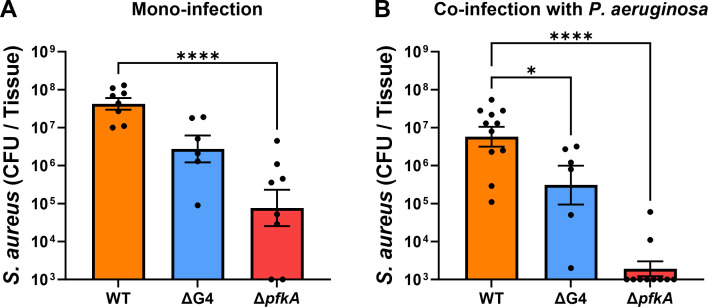
*S. aureus* glycolysis is required for increased dissemination into tissues during diabetic infection. Wild-type, ΔG4, or Δ*pfkA S. aureus* colonies recovered from the tissue surrounding catheters inserted into diabetic mice 11 days after infection, either (**A**) alone or (**B**) co-infected with *P. aeruginosa*. Bars represent geometric mean and standard error. ANOVA with Dunnett’s test for multiple comparisons (to WT): *P < 0.05, ****P < 0.0001.

To determine if the glucose-rich environment of the diabetic host was also contributing to increased *S. aureus* dissemination into the tissue, we analyzed infections with the ΔG4 and Δ*pkfA* mutant *S. aureus* strains in diabetic mice (Fig. 6). The Δ*pfkA* mutant was significantly inhibited in its ability to disseminate into the tissues during mono-infection, with the ΔG4 mutant displaying a strong trend for decreased dissemination (*P* < 0.06) compared to WT *S. aureus* (Fig. 6A). In co-infection with *P. aeruginosa*, significantly less ΔG4 and Δ*pfkA* mutant colonies were recovered from the tissue than WT *S. aureus* (Fig. 6B). Colonies were detected in tissue from only 2 of 10 mice infected with the Δ*pfkA* mutant. These trends of ΔG4 and Δ*pfkA* mutant *S. aureus* displaying decreased dissemination into the tissue compared to WT *S. aureus* were also observed to a lesser extent in normal mouse infections, including co-infection with *P. aeruginosa* (Fig. S4). Indeed, only the difference in dissemination between the Δ*pfkA* mutant and WT *S. aureus* during co-infection with *P. aeruginosa* reached the level of statistical significance. These experiments revealed that, while both glucose uptake and glycolysis likely support dissemination of *S. aureus* into the tissue, these processes are required for the increased dissemination afforded by the hyperglycemic environment of the diabetic host, especially in the presence of *P. aeruginosa.*

### *S. aureus* inhibits *P. aeruginosa* growth in co-infection by a glucose-dependent mechanism in a normal host, but a glucose-independent mechanism in a diabetic host

**Fig 7:**
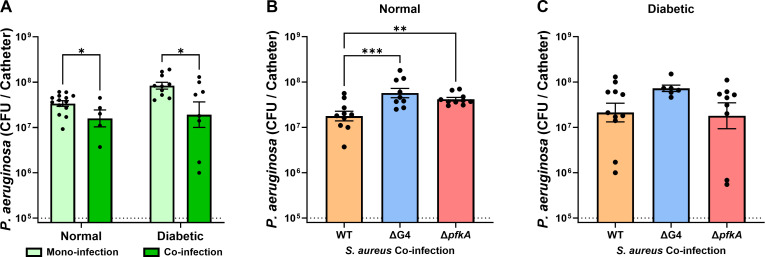
*S. aureus* inhibits *P. aeruginosa* growth in co-infection by a glucose-dependent mechanism in a normal host but a glucose-independent mechanism in a diabetic host. (**A**) *P. aeruginosa* colonies recovered from the catheter 11 days after insertion, either alone or in co-infection with wild-type *S. aureus*. Infections were carried out in normal and diabetic mice. (**B and C**) *P. aeruginosa* colonies were also recovered from the catheter after co-infection with ΔG4 or Δ*pfkA* mutant *S. aureus.* Bars represent geometric mean and standard error. Dotted line represents 10^5^ CFU catheter inoculum. ANOVA with (**A**) Tukey’s or (**B and C**) Dunnett’s test for multiple comparisons (to WT): *P < 0.05, **P < 0.001, ***P < 0.001.

While *S. aureus* growth in catheter infections was inhibited during co-infection with *P. aeruginosa*, we also observed that *P. aeruginosa* growth was moderately inhibited during co-infection as well (Fig. 7A). The amount of *P. aeruginosa* recovered from the catheter was not significantly different between a normal or diabetic host, and this remained true in both mono-infection and in co-infection with *S. aureus*. Thus, co-infection with *S. aureus* inhibited *P. aeruginosa* growth in the catheter independently of the diabetic host environment.

During co-infection of *P. aeruginosa* with the ΔG4 and Δ*pfkA* mutant *S. aureus* strains, the *P. aeruginosa* burden was significantly higher than in co-infection with WT *S. aureus*. (Fig. 7B). This provided evidence that the inhibition of *P. aeruginosa* growth by *S. aureus* observed in the catheter was a process that required both maximal glucose uptake potential and functional glycolysis by *S. aureus*. Surprisingly, however, *P. aeruginosa* burden within the catheter was not significantly different in co-infection with *S. aureus* mutants in a diabetic infection despite the higher glucose availability (Fig. 7C). This result suggested that *S. aureus* inhibition of *P. aeruginosa* growth during co-infection in a diabetic host was independent of *S. aureus* glucose uptake and metabolism.

### *P. aeruginosa*-secreted soluble factors inhibit *S. aureus* growth *in vitro* but not *in vivo*

**Fig 8:**
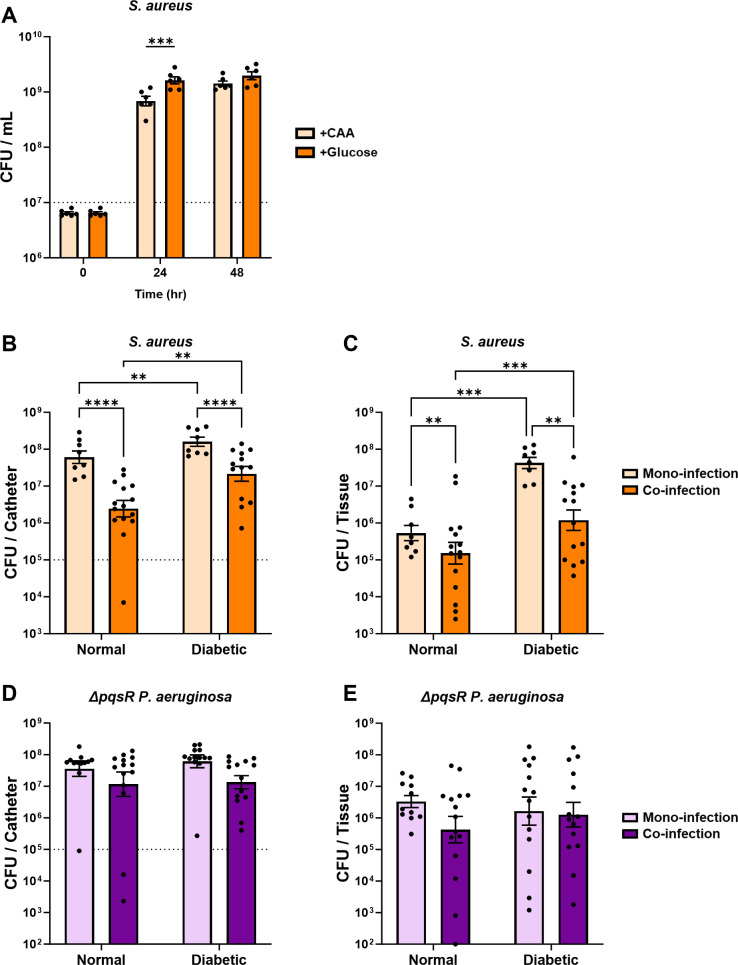
*P. aeruginosa* secreted factors under *pqsR* regulation inhibit *S. aureus* growth *in vitro* but not in the catheter model. (**A**) *S. aureus* was cultured *in vitro* in the presence of Δ*pqsR P. aeruginosa* culture supernatant supplemented with or without 25 mM glucose. All supernatants were fortified with 1% casamino acids before inoculation with *S. aureus.* Supernatants without glucose were supplemented with an additional carbon-equivalent of casamino acids. (**B and C**) *S. aureus* and (**D and E**) Δ*pqsR P. aeruginosa* CFU recovered from the catheter and surrounding tissue 11 days after initial infection, either alone or in co-infection. Infections were carried out in mice treated with streptozotocin (diabetic) or untreated mice (normal). Bars represent geometric mean and standard error. Dotted line represents inoculum. ANOVA with (**A**) Bonferroni’s or (**B–E**) the Tukey’s test for multiple comparisons: **P < 0.01, ***P < 0.001, ****P < 0.0001.

The inhibition of *S. aureus* growth by spent *P. aeruginosa* supernatant (Fig. 2) was consistent with numerous studies showing that *P. aeruginosa* secreted factors inhibit *S. aureus* growth (28, 30, 34, 35). These antistaphylococcal factors, which include pyocyanin, HQNO, and rhamnolipids, are positively regulated by the PQS QS system (20 – 23). We used a *P. aeruginosa* Δ*pqsR* mutant, which does not produce a majority of *P. aeruginosa* antistaphylococcal factors, to test our hypothesis that *P. aeruginosa* secreted factors were primarily responsible for *S. aureus* growth inhibition. Over the span 48 h, *S. aureus* was able to grow well (~2 log-fold) in spent supernatant from the *P. aeruginosa* Δ*pqsR* mutant both with and without supplemented glucose (Fig. 8A). Though *S. aureus* displayed marginally higher growth after 24 h when supplemented with glucose, no significant difference in *S. aureus* culture density was observed by 48 h. These results indicated that *S. aureus* growth inhibition when grown in *P. aeruginosa* spent supernatant was due to PQS-regulated secreted factors.

We hypothesized that the *P. aeruginosa* Δ*pqsR* mutant would not inhibit *S. aureus* growth during co-infection in the catheter model based on our *in vitro* results. Contrary to our hypothesis, the *P. aeruginosa* Δ*pqsR* mutant inhibited *S. aureus* growth in the catheter lumen and dissemination to surrounding tissues in both normal and diabetic mice (Fig. 8BC). These results suggest that although *P. aeruginosa* secreted factors can potently inhibit *S. aureus* growth *in vitro*, these secreted factors were not responsible for *S. aureus* growth inhibition in the catheter. We observed a trend of growth inhibition by *S. aureus* on the *P. aeruginosa* Δ*pqsR* mutant within the catheter, but it did not reach the level of statistical significance (Fig. 8D). There was no statistically significant effect on *P. aeruginosa* Δ*pqsR* mutant dissemination into the tissue by either the diabetic environment or co-infection with *S. aureus* (Fig. 8E).

## DISCUSSION

In this study, we employed a novel murine indwelling device co-infection model to examine the interactions between and virulence potential of *S. aureus* and *P. aeruginosa* in normal and diabetic infection microenvironments. We previously showed that *S. aureus* burden during SSTI in a diabetic host is significantly increased (10). Here, we show that *S. aureus* burden within the catheter was significantly increased (*P* < 0.0001) in a diabetic host (Fig. 1). Furthermore, the *S. aureus* burden was significantly increased in the tissue surrounding the catheter for diabetic animals (Fig. 5A). This is consistent with the increased dissemination and lesion size that is associated with *S. aureus* in diabetic SSTI (10). The greater dissemination of *S. aureus* into diabetic tissues observed here was associated with glucose uptake and glycolysis (Fig. 6). *S. aureus* dissemination in diabetic SSTIs is associated with both toxin and protease expression, which are under the control of the accessory gene regulator (Agr) system, with Agr activity being upregulated in response to increased glucose levels (10). It is likely that Agr-associated increases in *S. aureus* virulence factor production are similarly involved in the greater tissue dissemination observed here in the catheter model.

*P. aeruginosa* dissemination into the tissue during catheter infection was greater in diabetic animals (Fig. 5B). *P. aeruginosa* can use glucose as a sole carbon source (37, 38) but will preferentially use other sources (39). Therefore, while *S. aureus* glucose metabolism was required for increased tissue dissemination in the diabetic host, *P. aeruginosa* may respond to the hyperglycemic environment independently of its glucose metabolism. Despite the metabolic preferences of *P. aeruginosa*, there is evidence that diabetic hyperglycemia can contribute to *P. aeruginosa* virulence. It has been shown that high levels of glucose can increase *P. aeruginosa* biofilm formation (40), and it has been recently shown that *P. aeruginosa* isolated from patients with diabetes displays enhanced virulence potential in the presence of glucose (41). Another possible explanation for the increase in tissue dissemination is that immune suppression in the diabetic host allows for greater *P. aeruginosa* and *S. aureus* proliferation in the absence of immune pressure. In particular, *P. aeruginosa* is sensitive to free radical-mediated killing by phagocytes (42), a process which is suppressed in a diabetic environment (10). Indeed, others have shown in a mouse model of *S. aureus* and *P. aeruginosa* acute lung co-infections that *S. aureus* can prevent phagosome acidification through the production of α-toxin, thereby potentiating *P. aeruginosa* growth and virulence (43). Whether the increased glucose availability in the diabetic host contributes to increased *P. aeruginosa* virulence or how diabetic-induced immune suppression influences the polymicrobial infection of *S. aureus* and *P. aeruginosa* is yet to be elucidated.

The glucose concentration used in the *in vitro* experiments described here was 25 mM (450 mg/dL), representing a moderately hyperglycemic diabetic host. Mice in our *in vivo* experiments had blood glucose concentrations ranging from 300 to 600 mg/dL (16.7–33.3 mM). Additionally, glucose was not replenished *in vitro*, eventually being depleted by the cultured bacteria. However, glucose and other nutrients are constantly replenished in an *in vivo* environment by the host circulatory system. Glucose availability may be scarce in the catheter due to high bacterial concentrations in an environment with limited space and may also act as a physical barrier from host nutrient replenishment. However, it was determined that ~4-fold more glucose was available in the diabetic catheter environment (Fig. S1). As both glucose uptake and glycolysis were implicated in increased *S. aureus* dissemination during diabetic catheter infection (Fig. 4), *S. aureus* dissemination is most likely dependent on constant glucose availability in the subcutaneous tissue, which has previously been determined to be significantly increased during *S. aureus* infection in diabetic mice (10).

*S. aureus* glycolysis was crucial to its survival in the host, even in mono-infection, as the Δ*pfkA* mutant was attenuated in mono-infection (Fig. 4A; Fig. S2A) and displayed growth defects in culture (Fig. 3B). It is plausible that glycolysis does not confer unique resistance to *S. aureus* against *P. aeruginosa*-mediated growth inhibition but instead is required for continued growth potential despite the presence of *P. aeruginosa* when glucose is readily available. This explanation is supported by the fact that an *S. aureus* mutant lacking dedicated glucose transporters, but still able to perform glycolysis, did not show a defect in resistance to *P. aeruginosa*-mediated inhibition (Fig. 4C). An important consideration is that *S. aureus* lacking its four dedicated glucose transporters can still import glucose but at a slower rate (15). Presumably, this import is through other promiscuous sugar transporters. Additionally, *S. aureus* has transporters for other glycolytic substrates that may be available in the host environment (15). Thus, an *S. aureus* mutant deficient in the glycolytic enzyme that represents a bottleneck in the glycolytic process (the Δ*pfkA* mutant used here) serves as an important tool to study the role of *S. aureus* glycolysis during co-infection with *P. aeruginosa*. Interestingly, the Δ*pfkA* mutant showed decreased growth in the presence of a glucose-rich environment, mostly notably when grown in supernatants supplemented with glucose, either with or without the presence of *P. aeruginosa*-secreted factors (Fig. 3B). This is most likely caused by the accumulation of intracellular fructose-6-phosphate (36). The Δ*pfkA* mutant was also significantly killed during co-infection with *P. aeruginosa in vivo* (Fig. 4). These results imply that the *S. aureus* response to a glucose-rich environment is detrimental if glycolysis is inhibited and strongly indicate that glycolysis is required for resistance to *P. aeruginosa*-mediated growth inhibition.

In both *in vivo* and *in vitro* experiments with WT *S. aureus* and WT *P. aeruginosa*, diabetes or addition of glucose to the environment only conferred partial resistance of *S. aureus* to growth inhibition by *P. aeruginosa*, with *S. aureus* growth never completely reaching the level of *S. aureus* in the absence of *P. aeruginosa* or *P. aeruginosa*-related factors (Fig. 1 and 2). These data suggest that there are additional *P. aeruginosa*-associated inhibitory factors other than the ones that can be ameliorated by increasing *S. aureus* glycolysis.

Our study provides insight into the glycolysis-dependent mechanism by which *S. aureus* resists *P. aeruginosa*-mediated inhibition in a diabetic environment, but the specific mechanisms by which glycolysis confers resistance to *P. aeruginosa*-mediated factors are not fully understood. The *in vitro* assay results from WT and mutant *S. aureus* grown in *P. aeruginosa* culture supernatant (Fig. 2 and 3) followed a close trend with the results from the *in vivo* experiments (Fig. 1 and 4; Fig. S2) in terms of *P. aeruginosa* inhibition of *S. aureus* growth during co-infection in the catheter model. PQS-regulated secreted factors are known to inhibit *S. aureus* growth *in vitro* (30, 34, 35, 44 – 46). This is why we initially suspected that *P. aeruginosa* secreted factors could be responsible for inhibition of *S. aureus* growth in our *in vivo* model. *S. aureus* requires glycolysis and subsequent fermentation to overcome oxidative stress (17). When glucose was abundant in our *in vitro* assays, presumably *S. aureus* was able to overcome respiratory inhibitors produced by *P. aeruginosa*. However, *P. aeruginosa* Δ*pqsR* was still readily able to inhibit *S. aureus* growth in the catheter model (Fig. 8A and B). This suggests that *P. aeruginosa* factors other than secreted antistaphylococcal agents are responsible for the growth inhibition observed in the catheter model. One possibility is that *P. aeruginosa* secretes other non-PQS-regulated antistaphylococcal toxins *in vivo* that are not secreted *in vitro*. Another possibility is that *P. aeruginosa* outcompetes *S. aureus* for limited nutrients in a normal environment, but this pressure is alleviated in a diabetic microenvironment that is replete with glucose. Yet another potential explanation is that *P. aeruginosa* alters the immune response during normal infection in a way that influences *S. aureus* survival within the host. We recently demonstrated that the innate immune response is repressed in a diabetic infection (10). We predict that it is likely a combination of these factors. Future work will aim to establish the exact mechanism for *P. aeruginosa* suppression of *S. aureus* in normal infection and how *S. aureus* resists this suppression in diabetic infection.

In summary, we describe an *in vivo* model that provides useful mechanistic examination of *S. aureus* and *P. aeruginosa* co-infections. Importantly, the described model shares multiple similarities to chronic infections observed in the clinic. Emergence of *P. aeruginosa* infection often occurs in existing chronic infections in patients with CF or diabetic wounds where *S. aureus* already resides and *P. aeruginosa* can establish as a major (or even the dominant) infecting microbe (31, 47, 48). Relevant to this fact, our co-infection model mimics the emergence of a *P. aeruginosa* infection during an established *S. aureus* infection (Fig. 1). These co-infections also showed that *P. aeruginosa* was able to negatively influence *S. aureus* burden and dissemination (Fig. 1 and 5). Additionally, this model showed that *in vitro* interactions between *S. aureus* and *P. aeruginosa* shown by us and others do not always translate to an *in vivo* infection. Our *in vitro* experiments revealed that secreted factors from *P. aeruginosa* can inhibit *S. aureus* growth (Fig. 2), as many others have previously shown (34, 35, 44 – 46). We were also able to recapitulate a glucose-dependent method by which *S. aureus* was able to inhibit *P. aeruginosa* growth *in vivo* (Fig. 7), which has been demonstrated by others *in vitro* (49). However, we provide evidence that these well-established *in vitro* mechanisms do not provide an explanation for *in vivo* inhibition within the catheter model. While others have been able to model *S. aureus* and *P. aeruginosa* co-infections in rodent models (29, 43), the model described here allowed the study of *S. aureus* and *P. aeruginosa* co-infection both in a diabetic environment and using easily accessible, ordinary laboratory mice. These key distinctions led to the finding that the hyperglycemic environment of the diabetic host led to increased *S. aureus* survival and virulence despite *P. aeruginosa*-mediated inhibition, which may explain why *S. aureus* and *P. aeruginosa* co-infections are more common in patients with CFRD (28). Future efforts will apply this model to further validate *in vitro* results as well as continue to elucidate the mechanisms of polymicrobial interactions *in vivo.*

## MATERIALS AND METHODS

### Animals

Six- to eight-week-old male C57BL/6 mice were obtained from the Jackson Laboratory. Mice were kept at the University of North Carolina and used for experiments in accordance with an IACUC-approved protocol.

### Bacterial strains and mutants

All *S. aureus* strains used here were on the LAC background, including two previously described *S. aureus mutants:* a quadruple mutant lacking the four glucose transporters *glcA, glcU, glcB,* and *glcC* (ΔG4) (10, 15) and an upper glycolysis mutant lacking *pfkA*, which encodes phosphofructokinase (Δ*pfkA*) (10, 17). The *P. aeruginosa ΔpqsR* mutant was generated in the MPAO1 genetic background used in this study by allelic exchange as previously described (50) using the previously published deletion vector, pΔpqsR-suc (51).

### *In vivo S. aureus* and *P. aeruginosa* co-infection model

Subcutaneous catheter insertions were performed similar to what has been previously described (27). A 1-cm section of a 14G catheter was subcutaneously inserted into either flank of each mouse. Immediately following insertion, 1 × 10^5^ CFU of *S. aureus* (LAC strain) were injected into the catheter in 20-µL PBS. *S. aureus* infection was allowed to establish for 4 days before co-infection with 1 × 10^5^ CFU of *P. aeruginosa* (MPAO1 strain) in 20-µL PBS. *P. aeruginosa* mono-infections were also established at this time in catheters not previously inoculated with *S. aureus.* On day 11 following catheter insertion (7 days following co-infection with *P. aeruginosa*), catheters and immediately surrounding fibrotic tissue were removed, separated, and homogenized in 500-µL PBS. Homogenates were plated on mannitol salt agar (Sigma-Aldrich) and *Pseudomonas* isolation agar (Thermo Fisher Scientific) to isolate *S. aureus* and *P. aeruginosa* colonies, respectively. Glucose measurements from filtered catheter homogenates were made using a Glucose (GO) Assay Kit (Sigma Aldrich).

### Generation of diabetic mice

Immediately following *P. aeruginosa* infection on day 4, mice were made diabetic by intraperitoneal injection of streptozotocin (Sigma-Aldrich; 200–250 mg/kg) as previously described (10). Blood glucose was monitored starting on day 7. Injected mice with blood glucose <300 mg/dL by the end of the study were not considered diabetic and were removed from analysis.

### *In vitro S. aureus* growth in *P. aeruginosa* culture supernatant

Overnight cultures of *P. aeruginosa* or wild-type *S. aureus* were grown in 5 mL Luria-Bertani (LB) broth or tryptic soy broth (TSB), respectively, at 37°C, 225 rpm for 20 h. Next, 100 mL TSB without dextrose was inoculated with 1 mL of overnight broth culture in a pre-sterilized 500-mL Erlenmeyer flask. The Erlenmeyer flask opening was sealed with a Breathe-EASIER sealing membrane (Diversified Biotech) and incubated at 37°C, 270 rpm for 24 h. After incubation, the broth culture was decanted into 50-mL conical tubes and centrifuged at 3,200 × *g* for 30 min. This process of decanting and centrifugation was repeated with fresh 50-mL conical tubes, and then, supernatant was filter sterilized using a 0.45 µm filter. Filtered supernatant was evenly split into new containers. To one portion, glucose was added to 25 mM and Bacto casamino acids (Thermo Fisher Scientific) were added to 1% (wt/vol). The other portion was carbon-balanced with the addition of casamino acids alone to 1.385% (wt/vol). *S. aureus* supernatants were titrated to pH 7.15 using 10 M sodium hydroxide. Supernatants were then filter sterilized again and stored at −80°C for future use.

An overnight culture of *S. aureus* was prepared in TSB without dextrose. Ten microliters of the *S. aureus* overnight culture was added to 3 mL of *P. aeruginosa* or *S. aureus* supernatant supplemented with glucose and casamino acids or 3 mL *P. aeruginosa* or *S. aureus* supernatant supplemented with casamino acids only. Cultures were incubated at 37°C, 225 rpm, and bacteria were enumerated by measuring colony forming units (CFU) per mL after 0, 24, and 48 h post-inoculation using Brain Heart Infusion (BHI) agar drip plates.

### Statistical analysis

Power calculation on an initial pilot experiment of WT *S. aureus* mono-infection in normal mice established that a group size of *n* = 4 had statistical power of 0.8 to determine at least a twofold difference in CFU. Statistical comparisons between groups were made using ANOVA and performed using GraphPad Prism software. Corresponding *P*-values were calculated using Tukey’s or Dunnett’s post-test for multiple comparisons, where appropriate. Data expressed in CFU were log-transformed for statistical analyses.

## SUPPLEMENTAL MATERIAL

Supplemental file 1 spectrum.02299-23-s0001.docx Supplemental figures 1 to 4 and legends.

ASM does not own the copyrights to Supplemental Material that may be linked to, or accessed through, an article. The authors have granted ASM a non-exclusive, world-wide license to publish the Supplemental Material files. Please contact the corresponding author directly for reuse.
