# Supplementary material for: Hyperglycemia potentiates increased Staphylococcus aureus virulence and resistance to growth inhibition by Pseudomonas aeruginosa
Source: Microbiol Spectr. 2023 Nov 7;11(6):e02299-23. doi: 10.1128/spectrum.02299-23 (PMC10715105; doi:10.1128/spectrum.02299-23)
Supplement: Supplemental file 1 — Supplemental figures 1 to 4 and legends. [file spectrum.02299-23-s0001.docx]

**Figure S1:**

**Figure S1. Glucose availability during catheter infection.** Glucose concentration was measured inside the catheter after subcutaneous insertion and infection for 11 days with *S. aureus.* Measurements were made from catheters obtained from normal or diabetic mice. Bars represent mean and standard deviation. ***p* < 0.01, *t*-test.

**Figure S2:**

**Figure S2. ΔG4 and Δ*pfkA* *S. aureus* mutant growth in catheter co-infection with *P. aeruginosa* in normal mice*.*** Wildtype, ΔG4, or Δ*pfkA* *S. aureus* colonies recovered from catheters inserted into normal mice 11 days after infection, either **(A)** alone or **(B)** co-infected with *P. aeruginosa*. **(C)** Log-transformed difference in recovered *S. aureus* CFU from the catheter between mono-infection and co-infection with *P. aeruginosa*, representing inhibited growth by *P. aeruginosa.* Bars represent geometric mean and standard error. Dotted line represents 10^5^ CFU catheter inoculum. ANOVA with Dunnett’s test for multiple comparisons (to WT): ****p* < 0.001, *****p* < 0.0001.

**Figure S3:**

**Figure S3. *P. aeruginosa* dissemination into tissues during co-infection with *S. aureus* mutants.** *P. aeruginosa* colonies recovered from the surrounding tissue 11 days after catheter insertion and infection, either alone (-) or co-infected with WT, ΔG4, or Δ*pfkA* *S. aureus*. Infections were carried out in **(A)** normal or **(B)** diabetic mice. Bars represent geometric mean and standard error. ANOVA with Dunnett’s test for multiple comparisons (to *P. aeruginosa* mono-culture): **p* ≤ 0.05.

**Figure S4:**

**Figure S4. *S. aureus* mutant dissemination into tissue during infection in normal mice.** Wildtype, ΔG4, or Δ*pfkA* *S. aureus* colonies recovered from the tissue surrounding catheters inserted into normal mice 11 days after infection, either **(A)** alone or **(B)** co-infected with *P. aeruginosa*. Bars represent geometric mean and standard error. ANOVA with Dunnett’s test for multiple comparisons (to WT): ***p* < 0.01.

**Figure S1:**

**Figure S2:**

**Figure S3:**

**Figure S4:**
